# Characterising resting patterns of mother-calf humpback whale groups in a semi-enclosed embayment along the Australian east coast migration pathway

**DOI:** 10.1038/s41598-023-41856-1

**Published:** 2023-09-07

**Authors:** Alexandra Jones, Eleanor Bruce, Douglas H. Cato

**Affiliations:** 1 School of Geosciences, University of Sydney, Sydney, NSW 2006 Australia; 2 Marine Studies Institute, University of Sydney, Sydney, NSW 2006 Australia

**Keywords:** Ecology, Ocean sciences

## Abstract

On migration from low latitude breeding grounds to high latitude feeding grounds, humpback whale mothers and calves spend time resting in coastal embayments. Unlike other areas where resting has been documented, Jervis Bay, on Australia’s east coast, is remote from both breeding and feeding grounds, and provides a unique opportunity to compare resting behaviour observed within a semi-enclosed embayment to observations offshore. Land-based, and UAV surveys were conducted in Jervis Bay in 2018, 2019, and 2021. We show that (i) a disproportionately high percentage of groups with a calf enter Jervis Bay during the southbound migration, (ii) travelling speeds are significantly slower in the Bay compared to offshore, indicating resting behaviour, and (iii) aerial observations highlight resting and nurturing behaviour. Subsequently, we conclude that Jervis Bay is an important area for resting mother-calf humpback whale groups. Comparison with reports of resting behaviour during migration in areas nearer the breeding grounds shows commonalities that characterise resting behaviour in mothers and calves. This characterisation will allow improved monitoring and management of humpback whales in nearshore embayments during a critical stage of calf development, particularly those with increased anthropogenic activities.

## Introduction

Humpback whales (*Megaptera novaeangliae*) undertake one of the most extensive mammalian annual migrations between high-latitude summer feeding areas and low-latitude winter breeding and calving areas ^1–3^. This migration may be driven by the energetic benefits provided by the warm waters of breeding grounds that allow calves to conserve energy, leading to increased growth, development, and potentially future reproductive success ^2,4^. Travelling to warmer waters for whales to molt their skin has also been proposed as a factor potentially driving the migration^5^. During the migration, distinct temporal, and geographic separation of activities, namely breeding/calving, resting, and feeding, is linked to functional adaptations in resource availability ^6,7^. Humpback whales migrate along both the east and west coasts of Australia, to and from breeding grounds in the warm sheltered waters within the Great Barrier Reef on the east coast (International Whaling Commission (IWC) designated substock E1) ^6^ and on the North West Shelf on the west coast (substock D) ^8^. Whales are present inside the Great Barrier Reef from June to September ^6^. From here, the population migrates, ~ 5000 km south along a narrow migratory pathway ^9^ along the east Australian coastline to the nutrient rich Antarctic summer feeding grounds (IWC Antarctic Management Areas IV, V and VI). During the southbound migration females and their calves have been observed to rest in large open bays near the breeding grounds. Hervey Bay on the east coast ^10–12^ and Exmouth Gulf on the west coast ^13,14^. Resting has also been observed in open oceanic waters off Peregian Beach ^15^ on the east coast, however it is generally considered that mothers and calves prefer to rest in relatively shallow, calm waters or protected embayments ^16^. It has been proposed that these sheltered waters provide protection from rough seas, predators and conspecifics ^17^ and the calm surface conditions reduce energy consumption of lactating mothers and nursing calves ^18^.

The E1 subpopulation size was substantially reduced by commercial whaling, both from stations on the east Australian coast^19^ and from Soviet Union whaling ships in the Southern Ocean^20^. Whaling of this subpopulation ceased off the east coast in 1962 and in the Southern Ocean in 1968, but by then the population had been reduced to possibly as low as 100 individuals^19^. Since then there has been a substantial recovery of this subpopulation. Population size was estimated to be about 25,500 in 2015, increasing at about 11% per annum ^21^. The strong recovery of substock E1, has seen increased observations of humpback whales in coastal embayments along the southern migration pathway, including Hervey Bay, Queensland (QLD) ^10–12^ and Jervis Bay, New South Wales (NSW) ^22^.

Jervis Bay is a semi-enclosed embayment encompassing the multiple-use Jervis Bay Marine Park, waters of the Booderee National Park and part of the East Australian Defence Exercise Area, an important naval training site. In 1998, the area was declared a marine park by the NSW government based on its unique geology and oceanography, diverse habitats and ecosystems, and abundant flora and fauna. Humpback whales were abundant in Jervis Bay during the migration season prior to industrial whaling ^23^. Citizen science data collected from a commercial whale watching platform between 2007 and 2010 demonstrated the prevalence of mother-calf groups within the Bay from September to November corresponding to the southern migration ^22^. Whales, predominantly groups with calves, come into the Bay on the southbound migration thus geographically separating groups in the Bay from those migrating south, allowing for direct comparison of behaviour. Unlike other areas where resting has been documented previously, Jervis Bay is further from the recognised breeding grounds (~ 1500 km) almost one third of the way to the Antarctic feeding grounds which are ~ 3500 km further south. Although opportunistic feeding by sub-adult humpback whales has been observed at Eden, NSW ^24^, ~ 230 km south of Jervis Bay, this is linked to variable nutrient-rich upwelling events associated with the East Australian Current (EAC) and is unlikely to provide a consistent feeding area for energy intake by lactating mothers.

Previous studies have examined humpback whale movement trends and aggregations in breeding/resting grounds ^13^ and potential resting areas within migratory corridors ^10,11,25^, but there has been no systematic surveys of humpback whale usage patterns in semi-enclosed coastal embayments located at remote distances from breeding and feeding grounds. Off Australia, humpback whales follow the coastline during the migration ^26,27^. Due to the geomorphic configuration of Jervis Bay, access requires humpback whales to divert from the migration direction. Whales will enter Exmouth Gulf and Hervey Bay without needing to change their course of direction. However, whales will travel south past Jervis Bay in a southwest direction and to enter the Bay need to travel in a northwest direction. Jervis Bay has a much narrower entrance (3.7 km) which runs almost parallel to the migration compared with entrances of 70 km for Hervey Bay and 50 km for Exmouth Gulf, both of which run east to west and face the oncoming migration.

Resting opportunities involving low energetic expenditure are likely critical for minimising the rate of decline in body condition of lactating females ^13^ and optimising calf growth during key development stages which may have implications for individual reproductive success in adulthood ^2^. Additionally, in baleen whales calf growth is directly related to maternal energetic investment (milk transfer). Females with better body condition will produce larger calves who are stronger, faster and more resilient to environmental fluctuations (e.g., food shortages) ^28,29^. This has direct implications for surviving the migration back to colder polar feeding grounds.

Sheltered waters along the migration pathway, as in Jervis Bay, may facilitate nursing and energy conservation allowing calves to allocate energy to growth rather than movement ^30^. The fine-scale neonate humpback whale suckling behaviour of eight calves were quantified in Exmouth Gulf, Western Australia, and showed calves suckling 20.7 ± 7% of the total tagging time during which mothers were resting on the surface or submerged ^13,30^. Furthermore, these studies demonstrated that lactating mothers and their calves spent considerable time resting (~ 35%).

Jervis Bay is an area of significant naval, commercial, and recreational activity. Two commercial whale watching companies operate in the Bay and since 2019, permits have been approved for two commercial dive operators to offer swim-with-whale activities. Thus, with increasing numbers of resting whales, there is increasing potential for spatio-temporal overlap with anthropogenic activities which may impact on the energy conservation of lactating mothers and their calves.

Understanding how humpback whales use Jervis Bay is critical for guiding policy and management decisions regarding commercial, military, and recreational use of this area. This paper presents a characterisation of the behaviour of mother-calf groups resting in a semi-enclosed embayment, remote from the breeding grounds, where there is clear separation from the whales travelling past the bay on the migration. We compare results with observations in other areas in which humpback whales have been observed resting along the migration pathway to identify the characteristics of resting behaviour. The specific objectives were to: (i) determine the composition of humpback whale groups entering Jervis Bay during the southern migration using systematic surveys; (ii) compare the movement patterns of mother-calf groups, including speed and linearity, in Jervis Bay with whales travelling south on the main migratory pathway; (iii) quantify the behavioural states most frequently observed in the Bay; (iv) compare the results with those from other areas to determine general characteristics of resting behaviour. Land-based theodolite surveys and unoccupied aerial vehicles (UAVs) were employed during 2018, 2019 and 2021 to meet these objectives.

## Results

Land-based theodolite surveys were conducted on 23 (72%) of the scheduled survey days between 24 September and 25 October 2018, on 34 (89%) of the scheduled survey days between 30 September and 6 November 2019, and 14 (64%) of the scheduled surveys days between 6 and 27 October 2021.

### Group size and composition

A total of 609 humpback whales in 326 groups were observed entering/leaving, or within, Jervis Bay and 1955 humpback whales in 1181 groups, were observed travelling southwest past Point Perpendicular without entering the Bay. Humpback whale groups observed entering/leaving or within Jervis Bay, ranged in size from one to four whales (mean ± SD = 1.9 ± 0.7). Mother-calf pairs were the most frequently observed group composition (72%) followed by non-calf groups (15%), and mother-calf-escort groups (13%). Overall, 84% of groups that entered the Bay contained at least one calf. A total of 1181 groups were observed bypassing Jervis Bay, 22% of these groups contained a calf. A total of 507 groups containing a calf were observed, 247 of these (49%) entered/left Jervis Bay.

Three or more reliable fixes were obtained for 477 humpback whale groups. Of these, 158 groups were tracked entering/leaving, or within, Jervis Bay. Of these, 31 remained in the inshore area during observations (category 1), 57 groups entered/left the inshore area from offshore or the mouth of the Bay (category 2), and 70 groups entered/left the Bay but did not enter the inshore area (category 3). 319 groups were tracked offshore but did not enter the Bay. Groups were tracked on average for 44.3 min ± 45.9 (SD), the shortest track lasting for 10 min and the longest 247 min.

### Speed and linearity of movement

Mother-calf pairs tracked in the inshore area only (category 1), had significantly slower average track speeds (Mann–Whitney U = 247, *Z* = − 5.015, *p* < 0.001, 2-tailed), average net speeds (Mann–Whitney U = 95, *Z* = − 6.309, *p* < 0.001, 2-tailed), and lower linearity measurements (Mann–Whitney U = 86, *Z* = − 6.386, *p* < 0.001, 2-tailed) compared to mother-calf pairs migrating south offshore (category 4). The comparison of movement parameters for the four track categories are summarised in Table 1.

**Table 1:** Summary of movement paraments of mother-calf humpback whale pairs with averages ± standard deviation (SD) using data pooled from land surveys conducted from the Point Perpendicular Lighthouse in 2018, 2019, and 2021. Track categories represented groups that were tracked; (1) entirely within the inshore area, (2) from offshore/mouth of Jervis Bay into the inshore area, (3) entering/leaving the entrance of the Bay, (4) offshore. “Track speed” is the sum of the distances between consecutive fixes for a track divided by the sum of times between fixes. “Net speed” is the linear distance between the first and last fixes of a track divided by the total time. “Linearity” is track distance divided by net distance.

	Track category			
	1	2	3	4
Sample size	25	38	51	76
Average track speed ± SD (km/h)	2.4 ± 1.3	3.5 ± 1.3	3.3 ± 1.3	4.6 ± 1.8
Average net speed ± SD (km/ h)	1.1 ± 0.9	2.7 ± 1.6	2.9 ± 1.4	4.3 ± 1.7
Average linearity ± SD	0.5 ± 0.3	0.6 ± 0.3	0.8 ± 0.3	0.9 ± 0.2

Assessment of the proportion of mother-calf pairs travelling in almost straight lines, displaying “strong” linearity > 0.95^31^, showed that 39% of groups entering/leaving, or within, the Bay displayed strong linearity compared to 68.2% of groups travelling offshore. For groups that entered Jervis Bay, 30.1% had a linearity score below 0.5, suggesting meandering behaviour, compared to 3.5% of groups that did not enter the Bay. Of the groups that were tracked only in the inshore area (category 1), one (< 5%) had a linearity > 0.95 while 52% were < 0.5.

**Figure 1:**
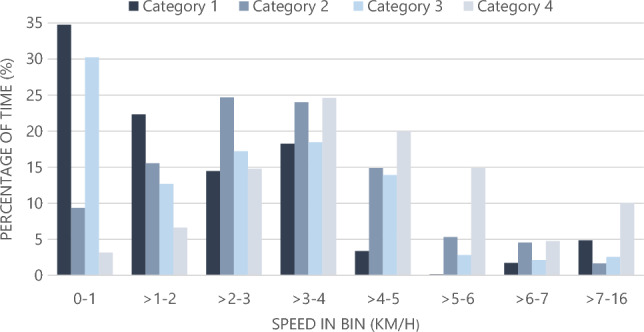
Frequency distributions of the percentage of time spent in speed classes grouped in bins of 1 km/h for mother-calf groups. Data taken from individual leg speeds (n = 1364). Bins from > 7 to 16 km /h were grouped given the low proportion of time spent at these speeds. Track categories represented groups that were tracked; (1) entirely within the inshore area, (2) from offshore/mouth of Jervis Bay into the inshore area, (3) entering/leaving the entrance of the Bay, (4) offshore.

Mother-calf pair groups spent considerably more time travelling at speeds < 1 km/h, indicating resting or drifting behaviour ^15^, within Jervis Bay compared to groups offshore (Fig. 1). Categories 1 and 3 demonstrated a clear preference (> 30% of the time) for travelling at speeds < 1 km/h. Whales in category 2 spent 48.7% of the time travelling at speeds 2–4 km/h. Offshore (category 4), whales showed a peak distribution of travelling at speeds 3.01–4 km/h (24.6% of the time). Offshore calf groups spent 3.2% of the time travelling at speeds < 1 km/h.

### UAV behaviour observations

**Figure 2:**
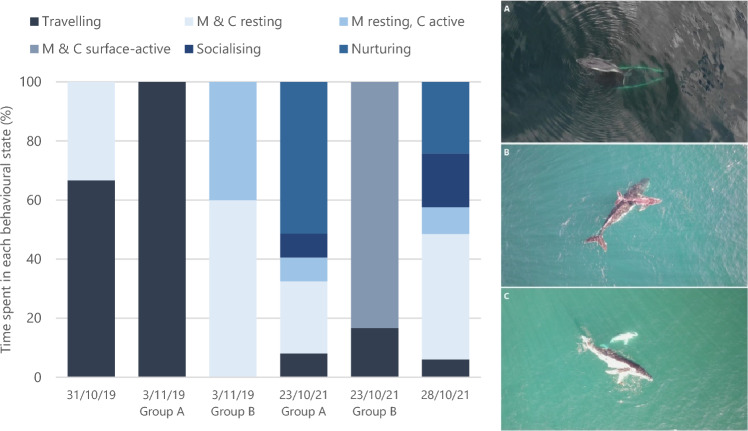
The proportion of time spent in behavioural states for six mother-calf humpback whale groups in Jervis Bay based on UAV aerial observations (left). M and C denote mother and calf, respectively. Humpback whales captured within Jervis Bay displaying: (**A**) resting, (**B**) nurturing, and (**C**) socialising behaviours (right).

UAV flights were conducted over four days during 2019 and 2021 (summarised in Table 2). The behaviour of six mother-calf groups were observed from UAV surveys (five mother-calf pair groups and one mother-calf-escort group) (Fig. 2).

**Table 2:** Summary of UAV flights conducted in 2019 and 2021. Dates where two groups were recorded denote an A and B group.

Year	Dates	Flights flown between	Flight time (to nearest min)	Duration of recorded behaviours (min)	Platform
2019	31 Oct	0805–0829	24	3	DJI Mavic 2 enterprise dual
	3 Nov	0715–1111	23	A: 10	
				B: 5	
2021	23 Oct	08011–0917	43	A: 37	DJI Mavic 2 enterprise advanced
				B: 6	
	28 Oct	1004–1316	33	33

Resting was the most observed behavioural state over the four days of flights (29% for both mother and calf resting, 9% when mother was resting and calf was active). This was followed by nurturing behaviour (29%), travelling (19%), socialising (10%), and surface-active behaviour (5%). 55% of the time spent ‘travelling’ were whales observed leaving the Bay. During 39% of observations, whales were completely submerged. All UAV flights were conducted in the inshore area.

## Discussion

This study used two complementary survey methods to provide detailed observations of humpback whales (E1 east coast population) in Jervis Bay, a site remote from the breeding grounds, during the southern migration during 2018, 2019, and 2021. The combination of (i) a high proportion of groups entering/leaving Jervis Bay containing a calf, (ii) a high proportion of time observed resting and (iii) aerial observations of resting and nurturing behaviour indicate that the area is a resting ground for mother-calf groups.

Over the observation period, 84% of humpback whale groups within Jervis Bay contained a calf. Of all groups that entered or left the inshore area (track categories 1 and 2), 92% contained a calf. Such a high proportion of groups with calves has not been observed elsewhere. Observing a high proportion of mother-calf groups towards the end of the southern migration is not unexpected. The migratory timing of humpback whales leaving the breeding and calving grounds is temporally staggered. Non-lactating females are the first to leave the breeding grounds, followed by immature males and females, mature males, and finally lactating females with their newly born calves ^1,11,32^. However, the proportion of groups within Jervis Bay containing a calf is considerably higher than other Australian areas where resting has been observed at equivalent seasonal timing. In Hervey Bay, Queensland, a peak occurrence of 40% of groups containing a calf was recorded from early August to mid-October ^10^. Similarly, the Exmouth Gulf, Western Australia, had an average of 41% for humpback whale groups containing a calf, peaking at 61% in mid-October ^33^. Off Peregian Beach, Queensland, the proportion of calf groups was 24% of all migrating groups over a similar time period ^15^. These calculations were taken of whales on the migratory corridor, indicating this is typical for whales offshore. Thus, the observations in Exmouth Gulf and Hervey Bay have a higher concentration than expected for the migration as a whole. Calf groups were about 22% of all groups passing Jervis Bay, similar to the proportion observed off Peregian Beach.

Mother-calf pairs inshore Jervis Bay (category 1) spent ~ 35% of the time resting at speeds < 1 km/h, consistent with 38% resting observed during UAV surveys (29% for mother and calf resting, 9% when mother was resting and calf was active). This is comparable with the 35% of time resting in Exmouth Gulf which was determined based on acceleration data from DTAGs ^13^, recognising that the different methods limit the accuracy of comparison. Aerial surveys over the Hawaiian breeding grounds observed that 26% of mother-calf pairs were resting ^34^. During the predominantly southbound migration off Peregian Beach, calf groups were observed to be drifting at < 1 km/h, apparently resting 16% of the time whereas non-calf groups drifted for 5.5% of the time ^15^. Results off Peregian Beach highlight resting behaviour in exposed oceanic waters, however, high proportions of mother-calf groups in embayments suggests these waters are preferred. Resting in exposed oceanic waters is likely during migrations when whales are moving from breeding areas (e.g., Hawaii or the South Pacific Islands) to feeding grounds in open waters rather than along coastlines.

The semi-enclosed embayment of Jervis Bay, with an irregular ellipsoidal shape, provides a distinct geomorphic setting along the eastern Australian coastline. A continuous stretch of ocean cliffs that form the coastal pathway between Beecroft Head and Cape St George is breached by the relatively narrow entrance of the bay (3.7 km). This discontinuity in the coastline is parallel to the south westerly direction of the migration and requires whales to detour from the main migratory pathway to move into the Bay. Although wind waves are generated across the surface of the bay, ocean swell (from the Tasman Sea and Southern Pacific Ocean) that pass through the entrance are increasingly refracted by a gently shelving bathymetry ^35^. The entrances to Hervey Bay and the Exmouth Gulf are oriented east–west facing towards the migrating whales and span close to 70 km and 50 km, respectively, each with a total area of ~ 4000 km^2^, so that whales of all group compositions are likely to move into these bays as they move south. This is consistent with the lower proportion of calf group observations in these locations than in Jervis Bay. The importance of Jervis Bay as an area for resting is further highlighted when considering the vast distances between both breeding (~ 1500 km) and feeding grounds (~ 3500 km).

Humpback whale mother-calf pairs in Jervis Bay travel at slower speeds, with less directed movements compared to groups travelling offshore on the southern migration. Travel speed and directional, linear movements offshore were as expected and consistent with other research along the Australian coast ^15,27^. Slow, non-linear movements, as observed by lactating females and their calves in Jervis Bay, suggest low energy expenditure and resting by these groups.

Resting implies saving of energy. For marine mammals, the rate of energy expended by travelling to overcome the drag resistance of water theoretically increases as the 3^rd^ power of their speed^36^. Total energy expenditure also includes the basal metabolic rate and factors involved in thermal regulation. Measurements of energy expenditure rate as a function of speed for a range of small marine mammal species generally show a gradual increase above the basal metabolic rate at the lowest speeds ^36,37,39^. With increasing speed, the energy expenditure increases at a progressively faster rate as the effect of travelling becomes more dominant. Hind and Gurney ^36^ provide a comprehensive model of the metabolic cost of swimming in marine homeotherms in which the basal metabolic rate dominates at very low speeds and the energy to overcome drag resistance dominates at high speeds, consistent with measurements. In this model, part of the basal metabolic rate at rest includes the generation of heat required to maintain thermal equilibrium of the body core if heat is lost to the surrounding colder water. Heat generated by travelling can to some extent compensate for this heat loss thus reducing the net additional energy expenditure during travel at low speeds. The result is that the increase in energy with speed from rest is more gradual than without this compensation, up to a speed where the heat from travelling exceeds that needed to compensate for heat loss to the water.

However, this effect may not be significant for large whales in warm waters, such as those in ^40^ and outside Jervis Bay where temperatures are typically 18–19 °C during the southbound migration. Modelling of thermal processes in marine mammals ^40–42^ has shown that large whales may need to dissipate excess heat in warm waters, especially when travelling, and that blood circulation through flippers, fins and flukes is important in dissipation. Energy saving through reduced speed may therefore be more for humpback whales in warm water than expected from the measurements for small marine mammals because there is not the need to generate heat at low speeds to compensate for heat loss to the water, as in the model of Hind and Gurney. With their rapid growth, calves have higher metabolic rates per kilogram of body mass than conspecific adults and higher than for adults of similar size from other smaller species^40^ so measurements of smaller marine mammal adults are not applicable, even if of similar size. There appear to be no measurements of the dependence of energy expenditure on speed for large whales in warm waters, or the data required to evaluate the model of Hind and Gurney to clarify this. However, it seems likely that there is considerable saving of energy by the significant reduction in speed and the high proportion of time spent with little movement by humpback whale mothers and calves in Jervis Bay compared to similar groups travelling offshore.

Respiration rate has been also be used to estimate energy expenditure in large whales ^14,43^. However, this assumes that expiration and inspiration gas exchange (tidal volume) is constant irrespective of speed or the rate of energy consumption. This is not supported by gas flow measurements at low speeds which found significant variation in tidal volume as flow rate of the blow as well as the duration varied and blows were observed to be weaker and more difficult to observe as the whales rested^44^. Hence respiration rates are likely to overestimate energy expenditure at low speeds and resting, leading to an underestimate of the dependence of energy expenditure on speed. In Jervis Bay, we found that blows from whales were often too weak to be reliably counted from the observation site, especially for calves and for longer distances, thus respiration rates were not measured. In studies off Peregian Beach, theodolite observations were found to underestimate respiration rates of migrating humpback whales compared with boat observation, especially the smaller blows from calves ^45^.

Bejder et al. ^13^ found that respiration rates of humpback whale mothers and calves in Exmouth Gulf were significantly less than for foraging whales off Greenland, based on aural detection of blows recorded on tags affixed to whales by suction cups. If respiration rate tends to overestimate energy expenditure at low speeds, the difference in energy expenditure between resting and foraging would be even more pronounced.

UAV surveys were conducted to observe fine-scale behaviour of humpback whales in Jervis Bay and complement movement parameters calculated using land-based theodolite data. Clear resting and nurturing behaviour was evident. In addition to the energetic advantages afforded by resting behaviour, as discussed above, maternal behaviour, including nursing and back riding, is linked to calf survival ^46^. Back-riding is particularly beneficial for younger calves by helping calves to stay afloat right after birth and facilitating protection from predators during developmental stages ^29,47^. Close proximity between mother and calf provides several advantages, including close access to milk, saving on energetic costs of travelling, reducing the need for loud acoustic communication which may attract predators ^30,47^. Few observations of surface-active behaviours in the UAV surveys further support minimisation of energy expenditure by mother-calf groups during their time in Jervis Bay.

UAVs provide a superior platform to visualise more subtle social and nurturing behaviours, as well as behaviours immediately below the surface, when compared to oblique methods from land or on a vessel which underestimate these activities ^48^. In our UAV surveys, whales were submerged for 39% of observations. Compared to boat-based observations, UAVs provide three times more observational capacity for the same time period ^49^. Additionally, UAV observation methods improve reliability of assigned behavioural states as video footage can be examined multiple times, by multiple researchers post-flight ^48^. The authors recognise the limitations associated with the restricted data available from four flights coordinated during periods of COVID related travel and fieldwork restrictions. However, these flights provided insights on nurturing and resting behaviour of submerged whales, that would not have been distinguishable from land or boat surveys.

This study confirms the role of Jervis Bay as a resting ground for mother-calf pairs from the east Australian humpback whale population as suggested by Bruce et al. ^22^. It also provides information that, when combined with other studies in the literature, allows a characterisation of resting behaviour that could be applied generally. Unlike other seasonally significant habitats for humpback whales, such as breeding and feeding grounds, requirements for an area to be considered a resting ground is less established in the literature. Jervis Bay meets the requirements of a resting ground as defined in other research studies in that it is an enclosed coastal area which provides shelter from open oceanographic conditions ^54^ where whales are not actively travelling and making up distance on their migration ^8^. We argue that these definitions are inadequate and not inclusive of all conditions and behaviours in which resting has been observed. Within the literature, it is accepted that resting behaviour involves whales near or on the surface displaying little activity other than breathing, also known as logging behaviour, but may also include calf back riding ^48,55,56^. Humpback whale mother-calf groups have been observed resting in exposed oceanic waters on the migration path ^15^, so resting is not confined to sheltered waters. An approach for improving the definition and characterisation of an area as a resting ground would be to conduct systematic surveys to quantify movements and observe behaviours over a significant proportion of the migration for more than one migration season. These could be directed towards open ocean areas to improve our knowledge of resting in non-enclosed and unsheltered waters. In this study we have demonstrated the benefits of using multiple survey methods to establish resting behaviour. This allowed validation across different data sets and showed the degree of complementarity between trends in the travel speed, linearity of movement and aerial UAV observations.

Resting mother-calf groups in Jervis Bay, and other sheltered waters, are especially vulnerable to human disturbances as they move at slow speeds and spend high proportions of time resting stationary at or near the surface ^13^. Despite being protected within the Jervis Bay Marine Park and Booderee National Park, mother-calf groups are temporally and geographically coincident with naval, recreational, and commercial activities during October, the peak time for their presence in Jervis Bay. Commercial whale watching and recently introduced swim-with-whale activities are also focused on observing these animals at close distances. This highlights the need to monitor and manage potential impacts of these activities during an important stage for calves undertaking their first southern migration.

## Materials and methods

### Field surveys

**Figure 3:**
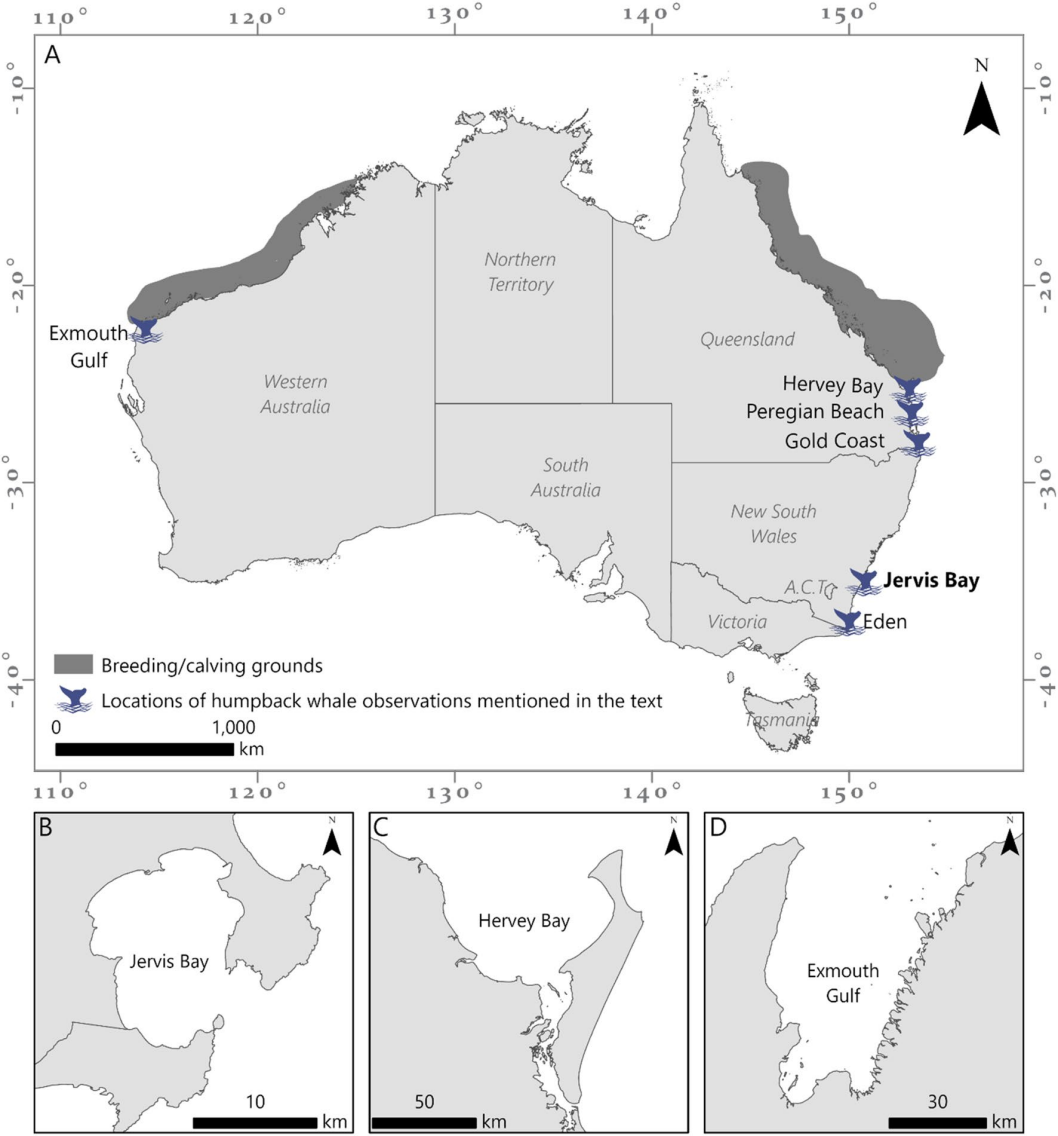
(**A**) Locations of documented aggregations for humpback whales migrating along the east and west coasts of Australia. A comparison of three areas where humpback whale resting has been observed off Australia is illustrated in (**B–D**). Figure created using ArcGIS Pro (v 2.9).

**Figure 4:**
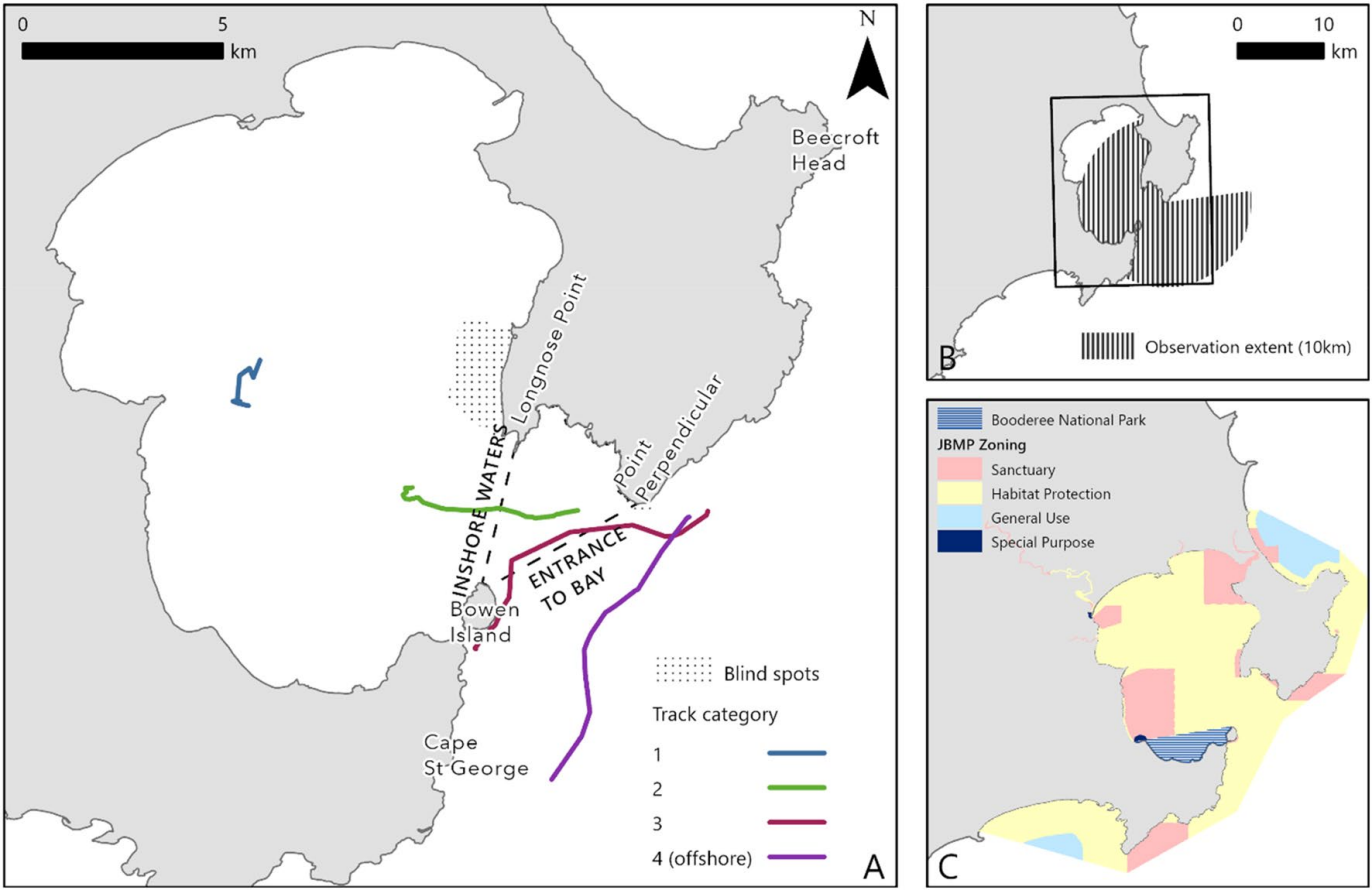
(**A**) Jervis Bay study site illustrating observation areas and example tracks to demonstrate movement from each category. (**B**) The 10 km observation extent from the Point Perpendicular Lighthouse. (**C**) The zoning and extent of the Jervis Bay Marine Park (JBMP) and the extent of the waters of the Booderee National Park. Figure created using ArcGIS Pro (v 2.9).

Fieldwork was undertaken in Jervis Bay (35°07′S, 150°42′E; Figs. 3 and 4), a semi-closed embayment on the NSW coastline, 115 km^2^ in area with an average depth of 15–20 m (maximum depth 40 m). Fieldwork was completed over three annual seasons; 24 September to 5 October, 2018; 30 September to 6 November, 2019; 4 October to 31 October, 2021. Restrictions resulting from the COVID-19 pandemic prohibited fieldwork in 2020. Survey timing and duration was based on the peak southern migration of humpback whales in the Jervis Bay area ^22^. Two survey methods were employed; daily land surveys and UAV surveys (summarised in Table 3). During all survey methods no signs of disturbance, as outlined in the scientific permits, were observed.

**Table 3:** Summary of field methods conducted during the 2018, 2019, 2021 southern migration seasons.

		2018	2019	2021
Land surveys	Survey hours	0800–1700	0800–1700	0900–1500
	Dates	28 Sept–5 Oct	30 Sept–6 Nov	4–31 Oct
UAV surveys	Dates	N/A	31 Oct, 3 Nov	23, 28 Oct

### Theodolite tracking

Point Perpendicular headland (75 m elevation), at the entrance of Jervis Bay, provided an optimal vantage point to observe whales entering or bypassing the Bay (Fig. 4). Operating from the Point Perpendicular Lighthouse upper balcony (~ 93 m above sea level), trained observers worked in teams of 3–4 on 2.5 h shift rotation with 1–2 dedicated spotters, 1 theodolite operator and 1 data entry operator, during daylight survey hours (Table 2). Spotters scanned the ocean and the Bay using the naked eye and 7 × 50 magnitude binoculars. Sighted whales were tracked using a theodolite (TC1105) connected directly to a laptop with custom software, VADAR© Version 2.0^57^, which determined the whale position using the horizontal angle (bearing) and the vertical angle to the whale to calculate the distance, as in previous experiments off Peregian Beach ^15,58^. If ≤ 5 whale groups were in the study area, all groups were tracked. For > 5 groups, groups within, entering, or leaving the Bay were prioritised. Consequently, to the best of our knowledge, most of the groups west of the entrance line (Fig. 4) were tracked. For groups offshore, we acknowledge some groups would have been missed.

Theodolite tracking was limited to whale groups within 10 km, the distance of sufficient visibility under good survey conditions (clear and calm). For a small area of the Bay (~ 2.8 km^2^), directly under the Point Perpendicular cliffs and behind (north of) Honeymoon Bay, observations were obscured by land (Fig. 4).

A whale group was defined as either a lone whale or multiple individuals (usually two or three) within 100 m of each other and surfacing at similar times. Following sighting, the group position was tracked at every surfacing event until they left the study area, travelled south offshore past Bowen Island, or were no longer detected. The group composition (e.g. lone adult; mother and calf (MC); mother, calf and escort (MCE)) was recorded for all sighted groups. A calf was defined as a whale of less than 70% the length of the accompanying whale, presumably its mother, with whom it maintained a close and constant association ^59^. Groups that merged or split were not included in the analysis.

### Sectioning of observation area, group composition and movement analysis

The observation area was divided into three sections (Fig. 4): (a) inshore Jervis Bay: the area west of the line from Bowen Island to Longnose Point, (b) the entrance of Jervis Bay: the area between the entrance line (west of the line from Bowen Island to Point Perpendicular) and area (a), and (c) offshore: all areas seaward of the line from Bowen Island to Point Perpendicular. The boundary of area (a) was derived using seabed exposure models ^60^ as a proxy for protection from ocean swell.

For the movement analysis, whale groups were assigned to four categories based on their tracked movements during the period of observation: category (1) groups whose tracks remained entirely within the inshore area, category (2) groups that were tracked from offshore or the mouth into the inshore area (including both tracks entering or leaving the inshore area), category (3) groups that entered or left the entrance without entering the inshore area, and category (4) groups that were tracked entirely offshore. The proportion of groups within the Bay were recorded and categorised by their composition.

Comparative analysis of whale movement patterns for the four categories was performed on time-enabled data points in ArcGIS Pro (version 2.9). Only tracks containing > 3 reliable theodolite position fixes and tracked for a total of duration > 10 min were included in the data sample. Track distance (m), duration (s), and speed (km/h) were calculated using ArcGIS Pro Motion Statistics.

The distance between any two consecutive fix positions is referred to as a leg. For each track the following parameters were calculated:

- Leg speed: the speed of each individual leg.
- Track speed: the swimming speed along a track calculated by summing the leg distances for entire track and dividing by the sum of the leg durations.
- Net speed: the straight line speed calculated by dividing the linear distance between the first and last position fixes in a track by the travel time between them (i.e. total duration of track);
- Linearity: a form of migration index, calculated by dividing the net distance covered over a track (i.e. the linear distance between the first and last fixes) by its cumulative distance (the sum of all leg distances). Linearity values range between 0 and 1, with values close to 1 representing a straight track-line, and values close to 0 indicating no constant direction.

Mann–Whitney-U tests were performed to compare the three movement parameters between mother-calf pairs in the inshore waters of Jervis Bay (category 1) and mother-calf pairs travelling south offshore (category 4). Mother-calf escort groups were excluded from these comparisons because the sample size in the bay/inshore was too small. Movement parameters from these two geographical areas could be assumed to be statistically independent which may not be the case for groups in categories 2 or 3. Non-parametric statistics do not assume normal data distribution and are less sensitive to unequal sample sizes ^61^.

### UAV surveys

UAV surveys were conducted using a DJI Mavic 2 Enterprise Dual (M2ED) in 2019, and a DJI Mavic 2 Enterprise Advanced (M2EA) in 2021. At the time of survey these were the most suitable light-weight UAV models available for launch and recovery on a small research boat. Following a confirmed whale sighting, whale’s behaviour and travel direction were observed for five minutes from a distance > 300 m at idle speed before the whales were approached whilst maintaining > 100 m distance. During flights the boat remained at this distance to provide a clear line of sight to the UAV and facilitate vertical positioning over the whales. The UAV was operated by the University of Sydney Chief UAV Pilot in accordance with Australian Civil Aviation Safety Authority regulations. A field researcher deployed and recovered the UAV by hand, assisted by a customised platform at the rear of the boat. An initial launch altitude of 55 m provided sensor field of view for whale detection within the video frame. The UAV pilot monitored the live video feed and once the whales were visible lowered to ≥ 25 m altitude. Video footage was captured at continuous 2–3 min intervals until the whales were no longer visible or travelling to a new location with direct movements or until 20% of the battery remained.

Jervis Bay is a restricted airspace and UAV flights are only approved during non-military use this confined the survey window to weekends and specified < 2 h time blocks on weekdays approved at short notice.

### UAV data analysis

Post-capture UAV video analysis of behavioural states followed methods outlined by Fiori et al. ^48^,^67^. Six mutually exclusive and cumulatively inclusive behavioural states were defined to describe whale group behaviour during each encounter: both mother and calf resting, mother resting whilst calf is active, travelling, surface-active, socialising, and nurturing (Table 4). Only behaviours observed were included in the analysis.

**Table 4:** Definitions of behavioural states of humpback whale groups modified from Fiori et al.^67^.

Both mother and calf resting	Mother and calf are motionless and horizontal at, or just below, the water’s surface, surfacing only to breathe
Mother resting, calf active	Mother is motionless and horizontal at, or just below, the water’s surface, surfacing only to breathe. The calf is displaying surface-active behaviours, including rolling, breaching, spy hopping, pectoral fin or head slapping
Travelling	Mother and calf are travelling from location to location with persistent, directional movement, covering noticeable distances
Surface-Active	Mother and calf are causing displacement of water at the surface by rolling, breaching, spy hopping, pectoral fin or head slapping. Behaviours in quick succession (< 1 min) but not necessarily simultaneous
Socialising	A whale mother or her calf are actively chasing or circling around the other whale
Nurturing	A whale mother and her calf are rubbing or touching; this includes mother lifting the calf with its rostrum. Suckling may be observed

Behavioural states were recorded at one-minute sampling intervals and assumed to remain constant between observations ^56^. If whales were last observed diving, travelling was the behavioural state allocated until the whales were resighted ^67^. The proportion of time that whales were fully submerged in the footage was also calculated.

### Ethics and permit statement

 Fieldwork activities were compliant with guidelines and regulatory requirements under permits authorization by the University of Sydney Animal Ethic Committee (permit 2019/1592), the Department of Primary Industries Marine Parks (permit MEAA19/179) and the Department of Planning, Industry and Environment, New South Wales (SL102287). Compliant with the Australian Civil Aviation Authority (CASA) all UAV flights were within visual line of sight. UAV flight approval within the Restricted Airspaces (R421A Nowra and R452 Beecroft Head) overlapping the Jervis Bay study site was obtained from the Australian Department of Defence.

## Data Availability

All datasets collected and analysed during the current study are available from the corresponding author on reasonable request.

## References

[1] Dawbin, W. The seasonal migratory cycle of humpback whales*.* in *Whales, Dolphins and Porpoises* (University of California Press, Berkeley, 1966).

[2] Rasmussen K (2007). Southern Hemisphere humpback whales wintering off Central America: Insights from water temperature into the longest mammalian migration. Biol. Lett..

[3] Stevick PT (2011). A quarter of a world away: Female humpback whale moves 10,000 km between breeding areas. Biol. Lett..

[4] Clapham P (2001). Why do baleen whales migrate? A response to Corkeron and Connor. Mar. Mamm. Sci..

[5] Pitman RL (2020). Skin in the game: Epidermal molt as a driver of long-distance migration in whales. Mar. Mamm. Sci..

[6] Smith JN (2012). Identification of humpback whale breeding and calving habitat in the Great Barrier Reef. Mar. Ecol. Prog. Ser..

[7] Owen K (2015). Effect of prey type on the fine-scale feeding behaviour of migrating east Australian humpback whales. Mar. Ecol. Prog. Ser..

[8] Jenner KC, Jenner MM, McCabe KA (2001). Geographical and temporal movements of humpback whales in Western Australian waters. APPEA J..

[9] Burns D (2014). Migratory movements of individual humpback whales photographed off the eastern coast of Australia. Mar. Mamm. Sci..

[10] Franklin T (2011). Seasonal changes in pod characteristics of eastern Australian humpback whales (*Megaptera novaeangliae*), Hervey Bay 1992–2005. Mar. Mamm. Sci..

[11] Franklin T, Franklin W, Brooks L, Harrison P (2018). Site-specific female-biased sex ratio of humpback whales (*Megaptera novaeangliae*) during a stopover early in the southern migration. Can. J. Zool..

[12] Franklin T (2021). Social behaviour of humpback whales (*Megaptera novaeangliae*) in Hervey Bay, Eastern Australia, a preferential female stopover during the southern migration. Front. Mar. Sci..

[13] Bejder L (2019). Low energy expenditure and resting behaviour of humpback whale mother-calf pairs highlights conservation importance of sheltered breeding areas. Sci. Rep..

[14] Ejrnæs DD, Sprogis KR (2021). Ontogenetic changes in energy expenditure and resting behaviour of humpback whale mother–calf pairs examined using unmanned aerial vehicles. Wildl. Res..

[15] Noad MJ, Cato DH (2007). Swimming speeds of singing and non-singing humpback whales during migration. Mar. Mamm. Sci..

[16] Ersts PJ, Rosenbaum HC (2003). Habitat preference reflects social organization of humpback whales (*Megaptera novaeangliae*) on a wintering ground. J. Zool..

[17] Smultea MA (1994). Segregation by humpback whale (*Megaptera novaeangliae*) cows with a calf in coastal habitat near the island of Hawaii. Can. J. Zool..

[18] Cartwright R (2012). Between a rock and a hard place: habitat selection in female-calf humpback whale (*Megaptera novaeangliae*) Pairs on the Hawaiian breeding grounds. PLoS ONE.

[19] Paterson R, Paterson P, Cato DH (1994). The status of humpback whales *Megaptera novaeangliae* in east Australia thirty years after whaling. Biol. Conserv..

[20] Clapham, P. *et al.* Catches of humpback whales, *Megaptera novaeangliae*, by the Soviet Union and other nations in the Southern Ocean, 1947–1973 (2009).

[21] Noad MJ, Kniest E, Dunlop RA (2019). Boom to bust? Implications for the continued rapid growth of the eastern Australian humpback whale population despite recovery. Popul. Ecol..

[22] Bruce E, Albright L, Sheehan S, Blewitt M (2014). Distribution patterns of migrating humpback whales (*Megaptera novaeangliae*) in Jervis Bay, Australia: A spatial analysis using geographical citizen science data. Appl Geogr.

[23] Dakin, W. J. *Whalemen Adventurers: The Story of Whaling in Australian Waters and Other Southern Seas Related Thereto, from the Days of Sails to Modern Times* (Angus & Robertson, 1938).

[24] Silva, I. *et al.* Mid-migration humpback whale feeding behavior off Eden, NSW, Australia. Report-International Whaling Commission (Report Number SC/63/SH12, 2013).

[25] McCulloch S, Meynecke JO, Franklin T, Franklin W, Chauvenet ALM (2021). Humpback whale (*Megaptera novaeangliae*) behaviour determines habitat use in two Australian bays. Mar. Freshw. Res..

[26] Paterson R, Paterson P (1989). The status of the recovering stock of humpback whales *Megaptera novaeangliae* in east Australian waters. Biol. Conserv..

[27] Gales, N. *et al.* Satellite tracking of Australian humpback (*Megaptera novaeangliae*) and pygmy blue whales (*Balaenoptera musculus brevicauda*). (2010).

[28] Christiansen F, Dujon AM, Sprogis KR, Arnould JP, Bejder L (2016). Noninvasive unmanned aerial vehicle provides estimates of the energetic cost of reproduction in humpback whales. Ecosphere.

[29] Christiansen F (2018). Maternal body size and condition determine calf growth rates in southern right whales. Mar. Ecol. Prog. Ser..

[30] Videsen SKA, Bejder L, Johnson M, Madsen PT, Goldbogen J (2017). High suckling rates and acoustic crypsis of humpback whale neonates maximise potential for mother-calf energy transfer. Funct. Ecol..

[31] Barendse J, Best PB (2014). Shore-based observations of seasonality, movements, and group behavior of southern right whales in a nonnursery area on the South African west coast. Mar. Mamm. Sci..

[32] Franklin, T. *The social and ecological significance of Hervey Bay Queensland for eastern Australian humpback whales (Megaptera novaeangliae)*, (2012).

[33] Irvine, L. & Salgado Kent, C. The distribution and relative abundance of marine mega-fauna, with a focus on humpback whales (2019).

[34] Herman LM, Antinoja RC (1977). Humpback whales in the Hawaiin breeding waters: Population and pod characteristics. Sci. Rep. Whales Res. Inst..

[35] Walker GT (1967). The Coastal Geomorphology of the Jervis Bay Area.

[36] Hind A, Gurney W (1997). The metabolic cost of swimming in marine homeotherms. J. Exp. Biol..

[37] Yazdi P, Kilian A, Culik B (1999). Energy expenditure of swimming bottlenose dolphins (*Tursiops truncatus*). Mar. Biol..

[38] Worthy GA, Worthy TA, Yochem PK, Dold C (2013). Basal metabolism of an adult male killer whale (*Orcinus orca*). Mar. Mamm. Sci..

[39] SchytteBlix A, Folkow LP (1995). Daily energy expenditure in free living minke whales. Acta Physiol. Scand..

[40] Lavigne D, Innes S, Worthy G, Edwards EF (1990). Lower critical temperatures of blue whales, *Balaenoptera musculus*. J. Theor. Biol..

[41] Ryg M (1993). Scaling of insulation in seals and whales. J. Zool..

[42] Hokkanen J (1990). Temperature regulation of marine mammals. J. Theor. Biol..

[43] Christiansen F, Rasmussen MH, Lusseau D (2014). Inferring energy expenditure from respiration rates in minke whales to measure the effects of whale watching boat interactions. J. Exp. Mar. Biol. Ecol..

[44] Sumich J, May M (2009). Scaling and remote monitoring of tidal lung volumes of young gray whales, *Eschrichtius robustus*. Mar. Mamm. Sci..

[45] Dunlop RA (2015). The behavioural response of humpback whales (*Megaptera novaeangliae*) to a 20 cubic inch air gun. Aquat. Mamm..

[46] Smultea, M. *et al.* in *Animal Behavior and Cognition* Vol. 4 1–23 (2017).

[47] Nielsen ML, Sprogis KR, Bejder L, Madsen PT, Christiansen F (2019). Behavioural development in southern right whale calves. Mar. Ecol. Prog. Ser..

[48] Fiori L, Martinez E, Bader MKF, Orams MB, Bollard B (2019). Insights into the use of an unmanned aerial vehicle (UAV) to investigate the behavior of humpback whales (*Megaptera novaeangliae*) in Vava'u, Kingdom of Tonga. Mar. Mamm. Sci..

[49] Torres LG, Nieukirk SL, Lemos L, Chandler TE (2018). Drone up! Quantifying whale behavior from a new perspective improves observational capacity. Front. Mar. Sci..

[50] Barendse J, Best PB, Carvalho I, Pomilla C (2013). Mother knows best: occurrence and associations of resighted humpback whales suggest maternally derived fidelity to a Southern Hemisphere coastal feeding ground. PLoS ONE.

[51] Sheehan, S. & Blewitt, M. in *Australian Marine Science Association (AMSA)* (Gold Coast, Queensland, Australia, 2013).

[52] Clapham PJ, Mayo CA (1987). Reproduction and recruitment of individually identified humpback whales, *Megaptera novaeangliae*, observed in Massachusetts Bay, 1979–1985. Can. J. Zool..

[53] Clapham PJ (1993). Seasonal occurrence and annual return of humpback whales, *Megaptera novaeangliae*, in the southern Gulf of Maine. Can. J. Zool..

[54] Braithwaite JE, Meeuwig JJ, Jenner KC (2012). Estimating cetacean carrying capacity based on spacing behaviour. PLoS ONE.

[55] Sprogis, K. R., Bejder, L. & Christiansen, F. Swim-with-whale tourism trial in the Ningaloo Marine Park, Western Australia (2017).

[56] Lundquist D (2013). Response of southern right whales to simulated swim-with-whale tourism at Península Valdés, Argentina. Mar. Mamm. Sci..

[57] Visual and Acoustic Detection and Ranging at Sea (VADAR) v. 2.0 (University of Newcastle).

[58] Cato DH (2013). A study of the behavioural response of whales to the noise of seismic air guns: Design, methods and progress. Acoust. Aust..

[59] Sprogis KR, Bejder L, Hanf D, Christiansen F (2020). Behavioural responses of migrating humpback whales to swim-with-whale activities in the Ningaloo Marine Park, Western Australia. J. Exp. Mar. Biol. Ecol..

[60] Geoscience, A. (data.gov.au, 2017).

[61] MacFarland TW, Yates JM (2016). Introduction to Nonparametric Statistics for the Biological Sciences Using R.

[62] Katona, S. *et al. *Identification of humpback whales by fluke photographs In *Behavior of Marine Animals* 33–44 (Springer, 1979).

[63] Craig, A., Herman, L. & Pack, A. Estimating residence times of humpback whales in Hawaii (2001).

[64] Calambokidis J (2001). Movements and population structure of humpback whales in the North Pacific. Mar. Mamm. Sci..

[65] Constantine R (2012). Abundance of humpback whales in Oceania using photo-identification and microsatellite genotyping. Mar. Ecol. Prog. Ser..

[66] Friday N, Smith TD, Stevick PT, Allen J (2000). Measurement of photographic quality and individual distinctiveness for the photographic identification of humpback whales, *Megaptera novaeangliae*. Mar. Mamm. Sci..

[67] Fiori L, Martinez E, Orams MB, Bollard B (2020). Using Unmanned Aerial Vehicles (UAVs) to assess humpback whale behavioral responses to swim-with interactions in Vava’u, Kingdom of Tonga. J. Sustain. Tour..

